# Comparative Analysis of Diet Quality, Iron Intake, and Supplementation Among Vegan and Omnivorous Amateur Runners Living in Urban Areas

**DOI:** 10.1002/fsn3.72088

**Published:** 2026-07-09

**Authors:** Gabriela Lewandowska, Hubert Dobrowolski

**Affiliations:** ^1^ Student Research Group “Supplement” VIZJA University Warsaw Poland; ^2^ School of Medical and Health Sciences VIZJA University Warsaw Poland

## Abstract

Plant‐based diets, including vegan and vegetarian patterns, are gaining popularity among physically active individuals, including amateur runners. While such diets may offer health benefits, they also carry a risk of inadequate intake of key nutrients, among which iron plays a crucial role. This study compared diet quality, iron intake, and dietary supplementation among vegan, lactovegetarian, and omnivorous amateur runners in Warsaw, Poland. One hundred runners (52 males, 48 females; aged 18–65) were classified into vegan (*n* = 36), lactovegetarian (*n* = 10), and omnivorous (*n* = 54) groups. Dietary intake was assessed via Food Frequency Questionnaire (FFQ) and IRONIC‐FFQ for iron, and diet quality was measured by the Pro‐Healthy Diet Index (pHDI). Nutritional knowledge was assessed using a 10‐item test, and supplement use was evaluated via proprietary self‐reported questionnaire. Group differences were evaluated with ANOVA, *t*‐test, and Kruskal–Wallis test. Pearson's *χ* ^2^ tests and Spearman correlation were also performed. No significant differences in age, Body Mass Index (BMI), height, or weight were found across groups. Plant‐based runners had higher median iron intakes (VEG: 20.5 mg/day; LOW: 19.97 mg/day) than omnivores (OMN: 13.21 mg/day, *p* < 0.001). pHDI was highest in the LOW group (31.82), followed by VEG (25.57) and OMN (24.76), with no significant sex differences. Supplement use was more frequent in plant‐based groups (VEG: 91.7%, LOW: 90.0%, OMN: 63.0%, *p* = 0.004); vitamin B12 and D were most common. Nutritional knowledge was significantly greater in VEG and LOW groups versus OMN (*p* = 0.002). Iron intake correlated positively with nutritional knowledge (*p* = 0.014), while pHDI correlated negatively with BMI (*p* = 0.002). Well‐planned plant‐based diets supported adequate iron intake and diet quality in amateur athletes, with increased supplement use and nutritional awareness mitigating deficiency risks. Nutrition education and individual nutrition planning are advised for all athletes, with attention to iron status, especially in female runners.

## Graphical abstract

In 100 amateur runners from Warsaw, Poland (vegan, lacto‐/ovo‐/pesco‐vegetarian, and omnivorous groups), plant‐based runners achieved dietary iron intakes comparable to or higher than omnivores (medians 20.5 and 19.97 vs. 13.21 mg/day; *p* < 0.001) and similar or better diet quality, as measured by the Pro‐Healthy Diet Index. Dietary supplement use (91.7% and 90.0% vs. 63.0%; *p* = 0.004) and nutritional knowledge were both significantly higher in the plant‐based groups. Overall, a well‐planned plant‐based diet can meet the nutritional needs of amateur runners. Supplement use and good nutritional awareness may help guard against iron deficiency, especially in female runners.

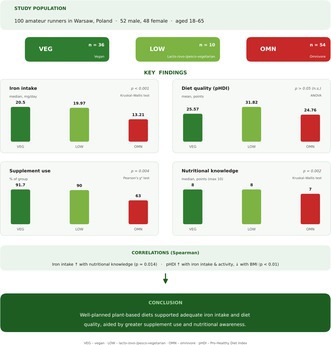

## 1 Introduction

Physical fitness and engaging in regular physical activity are one of the key aspects of public health and the prevention of many metabolic diseases (Anderson and Durstine 2019; Bull et al. 2020; Wahid et al. 2016). The importance of regular physical activity is frequently emphasized in numerous recommendations (Gelius et al. 2020; Piercy et al. 2018; Wirnitzer et al. 2016). One sport that enjoys widespread popularity is running, which has been associated with a reduced risk of all‐cause, cardiovascular, and cancer mortality (Lee et al. 2014). Therefore, such regular activities serve as an effective method of maintaining individual health. A vegan diet has gained popularity in recent years, especially among physically active people, such as runners (Rogerson 2017; Wirnitzer et al. 2022). Despite growing interest, there are still many questions regarding the impact of a vegan diet on diet quality of athletes, intake of selected nutrients, and the need for dietary supplementation (Tanous et al. 2024; Wirnitzer et al. 2021). A vegan diet, if composed inappropriately, may lead to limited intake of key nutrients such as high‐biological‐value protein, iron, calcium, vitamin B12, and vitamin D, which are crucial for adequate athletic performance (Bakaloudi et al. 2021). The current scientific evidence does not suggest that a vegan diet impairs athletic performance, training adaptation, or recovery in professional athletes (Isenmann et al. 2023).

Due to lack of proper knowledge on diet among amateurs, there is a risk of imbalanced diet, resulting in nutrient deficiencies, which in turn may negatively affect health and physical capacity (Wang et al. 2023). Therefore, monitoring the quality of the diet among athletes, including amateur and vegan athletes, seems justified. Interestingly, available studies suggest that plant‐based runners generally demonstrate comparable or higher diet quality than omnivores, with greater consumption of fruits, vegetables, and legumes (Nebl et al. 2019; Tanous et al. 2024; Wirnitzer et al. 2022). This raises the question of whether higher diet quality among plant‐based runners may be related to greater nutritional knowledge. Among athletes, nutritional knowledge has been found to be low to moderate, with gaps identified particularly in relation to micronutrient requirements and supplementation (Heaney et al. 2011; Hopper et al. 2025). To date, no study has specifically examined nutritional knowledge among plant‐based recreational runners, representing a clear gap in the literature that the present study aims to address.

Dietary supplementation has been identified as a key strategy among vegan runners to address potential nutritional gaps, including iron deficiency (Wirnitzer et al. 2021). Studies show that vegan endurance runners report more than two‐fold higher intake of vitamin supplements compared to omnivores, with vitamin B12, vitamin D, and magnesium being the most used (Wilson 2016). However, despite higher overall supplement use, not all vegan athletes appear to supplement the most critical nutrients, with one study reporting that only around half of physically active individuals following a plant‐based diet supplemented with vitamin B12 (Craddock et al. 2022).

One of the nutrients of particular interest among vegans and athletes is iron. Its intake plays a key role in metabolism and physical performance. It plays a crucial role in oxygen transport in the body, as it is a component of hemoglobin and myoglobin (Goswami et al. 2002). Deficiency may lead to reduced performance, fatigue, weakness, and other health issues (Kardasis et al. 2023; Pengelly et al. 2025). This is especially relevant among physically active individuals such as runners, for whom adequate iron levels can influence training effectiveness and overall fitness (Solberg and Reikvam 2023). Iron deficiency, with or without anemia, is more common in endurance athletes than in the general population (Król et al. 2026). This is particularly true for runners, who are at higher risk of iron deficiency due to several factors such as excessive sweating, gastrointestinal bleeding, urinary blood loss, hemolysis, and other mechanisms (Sinclair and Hinton 2005). Moreover, vegan runners may face greater challenges in absorbing iron, as plant‐based (non‐heme) iron is less bioavailable compared to heme iron found in animal products (Piskin et al. 2022). Additionally, increased iron loss through sweating, characteristic of runners, may be more problematic in a plant‐based diet where iron availability is limited (Damian et al. 2021). Therefore, adequate iron intake in vegan runners' diets is particularly important to ensure optimal performance, recovery, and overall health. Despite the lower bioavailability of non‐heme iron, iron intake in vegan runners has been shown to meet or exceed that of omnivores in some studies, though adequacy remains a concern, particularly among female athletes (Badenhorst et al. 2021; Holmes and Willoughby 2018; Luna et al. 2026).

Based on the existing literature, three hypotheses were formulated for this study. First, it was hypothesized that runners following plant‐based diets would demonstrate overall diet quality comparable to or higher than that of runners following an omnivorous diet. Second, it was hypothesized that, despite the lower bioavailability of non‐heme iron, plant‐based runners would show dietary iron intakes comparable to or higher than those of omnivores. Third, it was hypothesized that vegan runners would report more frequent use of dietary supplements compared to omnivores. Given the importance of physical activity for human health, the major role played by diet and iron in physical performance, and the potential risks of plant‐based diets in amateur athletes, the aim of our study was to conduct an analysis of diet quality, iron intake, and supplementation among vegan amateur runners compared to omnivorous runners residing in urban areas.

## 2 Methods and Materials

### 2.1 Study Participants

Due to the lack of accurate databases on the number of amateur runners in Poland and in individual cities, as well as the lack of data on their diets, it is difficult to estimate the minimum sample size. Assuming a population of 30,000 people (the number of participants in the Warsaw Half Marathon and accompanying races), a sample of 96 people would be sufficient for a 95% confidence interval and a maximum margin of error of 10%. However, this figure is rather imprecise, given that not all runners take part in road races, and organized running events also attract competitors living outside the race area. To clarify the sample size, a review of the literature was conducted, which found that a significant number of studies included samples of fewer than 100 people (Bykowska‐Derda et al. 2022; Śliż et al. 2021). It was therefore assumed that the minimum number of study participants is 100.

A total of 108 participants initially took part in the study. However, eight individuals were excluded due to not meeting the inclusion criteria or failing to provide complete responses or informed consent. As a result, the final study sample consisted of 100 amateur runners (52 male and 48 female), aged between 18 and 65 years. All participants resided in Warsaw, the capital city of Poland, representing a large urban population. The inclusion criteria comprised self‐declared residence in one of Warsaw's districts, age between 18 and 65 years, and engagement in amateur running activity. Exclusion criteria included professional athletic status, residence outside of Warsaw, being under 18 or over 65 years of age, injury in the last 3 months, and failure to complete the questionnaire or provide informed consent.

Approval for the study was obtained from the Research Ethics Committee of the University of Economics and Human Sciences in Warsaw (approval no. 03/02/20025, dated 27.02.2025). Each participant of the study was instructed about the tools and methods used in the study, the possibility of withdrawing from the study at any stage and gave informed consent to participate in the study. All methods were carried out in accordance with the Declaration of Helsinki.

Participants were classified into three dietary groups based on self‐reported eating habits: VEG—Vegan group, LOW—Lactovegetarian group (including individuals following pesco‐, lacto‐, ovo‐, or lacto‐ovo‐vegetarian diets), and OMN—Omnivore group. Group classification was verified using the Food Frequency Questionnaire (FFQ) included in part B of the *KomPAN* questionnaire administered by The Polish Academy of Sciences (Jeżewska‐Zychowicz et al. 2014), which assessed the participants' intake of specific food groups to ensure alignment with their declared dietary pattern.

### 2.2 Data Collection

Data were collected using a Computer‐Assisted Website Interview (CAWI) self‐reported questionnaire. Participants were recruited via social media platforms, primarily through social media groups dedicated to running communities in Warsaw, Poland. The invitation to participate included a direct link to the online survey and was addressed to adult individuals residing in urban areas of Warsaw. To confirm their location, participants were asked to indicate the district of Warsaw in which they resided. Before beginning the questionnaire, participants were informed about the aim of the study, the voluntary nature of participation, their right to withdraw at any stage, and the assurance of anonymity and data confidentiality. To participate in the study, individuals were required to provide informed consent by ticking a relevant statement before proceeding with the survey. Participants were classified into one of three dietary groups: vegan, lactovegetarian, or omnivorous, based on self‐reported dietary pattern, selected from three predefined options provided in the questionnaire and then verified using the FFQ questionnaire.

### 2.3 Questionnaire

The questionnaire consisted of 88 questions in total and was divided into four sections: Section 1—metric data, Section 2—diet quality and frequency of high iron food consumption, Section 3—assessment of nutrition knowledge, and Section 4—iron intake.

The first section of the questionnaire gathered demographic and anthropometric data. Demographic variables included age, sex, dietary habits (type of diet followed), presence of chronic diseases, and self‐assessed diet quality, nutrition knowledge, and financial situation. These self‐assessments were based on participants' own perceptions and were made using a 5‐point Likert scale. Anthropometric data included self‐reported height and body mass, from which Body Mass Index (BMI) was calculated. Additionally, the section included questions about the level of education, place of residence, which was used to verify residency within Warsaw, the average duration of exercise sessions, sources of dietary information, and the use of dietary supplements. Specifically, participants were asked whether they regularly consumed dietary supplements containing vitamin B12, iron, zinc, vitamin D, omega‐3, iodine, calcium, and protein. Physical activity was assessed with a single question— “How many times per week do you usually train?”—with five response categories: 0 times, 1–2 times, 3–4 times, 5–6 times, and more than 7 times per week.

Part B of the KomPAN questionnaire was used to determine diet quality and the frequency of consumption of selected food product groups (Jeżewska‐Zychowicz et al. 2014) This part originally includes 33 questions to evaluate the foods consumed over the past year during meals and snacks, both at home and away. However, the number of statements was reduced to 28 in this study, because “Non‐Healthy Diet Index” (nHDI), was not calculated in the study, since it contains many animal‐derived products, which are not present in any of plant‐based diets, including vegan, lactoovovegan, pescatarians, or even semi‐vegans and it would be inappropriate to compare groups with plant‐based diets with OMN group. The questions included frequencies of consumption of such products as meat/fish/eggs (6 questions), fruits/vegetables/legumes/potatoes (5 questions), cereals products (4 questions), dairy products (4 questions), drinks (4 questions), fats (3 questions), and other products (2 questions), which allow calculation of the “Pro‐Healthy Diet Index” (pHDI) as an indicator of diet quality in the study group.

The Food Frequency Questionnaire (FFQ) responses were recorded on a 6‐point scale, where participants indicated their frequency of consumption as: never, 1–3 times a month, once a week, several times a week, once a day, or several times a day. Numerical values were assigned to the responses as follows: several times a day = 2, once a day = 1, several times a week = 0.5, once a week = 0.14, 1–3 times a month = 0.06, never = 0. The total score range, therefore, was 0–20 points divided into three categories: low (0–6.66), moderate (6.67–13.33), and high (13.34–20), representing the intensity of pro‐healthy diet characteristics. The pHDI was calculated based on the following formula compiled by developers (Jeżewska‐Zychowicz et al. 2014)

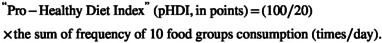




A higher Pro‐Healthy Diet Index (pHDI) score reflects a greater presence of eating habits that are considered beneficial to health, indicating a stronger alignment with pro‐healthy diet characteristics.

In Section 3 of the questionnaire a 10‐question test was used to assess the participants' nutritional knowledge. Six of these questions were taken from Part C of the KomPAN questionnaire (Jeżewska‐Zychowicz et al. 2014) that were most thematically linked to the nutrition of people on a plant‐based diet. In addition, a further 4 questions related to this topic were constructed. This allowed for a more detailed understanding of the beliefs and nutrition knowledge among VEG and LOW individuals. All questions are presented in Table 1. Participants could choose between 3 responses: “True,” “False,” and “Do not know.” For each correct answer (“True” or “False”), 1 point was assigned, and for an incorrect answer or “Do not know” answer, 0 points were assigned. Then, the points were added up.

**TABLE 1:** Selected questions assessing nutritional knowledge.

Question number	Question	Correct answer
1	Whole meal bread contains more fiber than white bread	True
2	Only children and adolescents should consume milk	False
3	Fruit and/or vegetables should be eaten at every meal	True
4	Frequent consumption of fatty sea fish accelerates the development of atherosclerosis	False
5	Vitamin D consumption is crucial for calcium absorption, as it aids its absorption in the intestines.	True
6	Eating fruits rich in vitamin C increases the absorption of iron	True
7	A consequence of a vegetarian diet is an increased risk of anemia	True
8	Vitamin B12 naturally occurs in plant‐based foods	False
9	Non‐heme iron, found in vegan foods, is less efficiently absorbed by the body than heme iron	True
10	Consuming sufficient omega‐3 fatty acids in a plant‐based diet is more challenging.	True

In Section 4 of the questionnaire, information on the iron intake was collected using the IRON Intake Calculation–Food Frequency Questionnaire (IRONIC‐FFQ), a tool previously validated for assessing dietary iron (Głąbska et al. 2017). This questionnaire has previously been used to assess the iron intake of both the general population and professional athletes (Dobrowolski and Włodarek 2020). In this questionnaire, iron intake can be evaluated based on declared consumption of specific food groups, including cereals, meat products, vegetables, nuts, fruits, cocoa‐based items, eggs, potatoes, dairy products, fats, and fish. Participants were asked to report the typical number of servings they consumed from each food group during a regular week over the past year. The questionnaire used open‐ended questions, allowing participants to indicate both whole and fractional servings. Study participants were instructed to include foods consumed directly as well as those added to prepared dishes.

For the analysis, weekly servings were divided by seven to estimate average daily intake. Iron intake from each food group was calculated using the following formula:

$$\begin{array}{c} \text{iron intake}\;\left(\mathrm{mg}\right)=\text{average daily number of servings}\\ \;\times \text{standard iron content}\;\mathrm{per}\;\text{serving} \end{array}$$

According to the calculation instructions indicated by the developers of the questionnaire (Głąbska et al. 2017).

### 2.4 Statistical Analysis

Statistical analysis was conducted using SPSS v. 29.0 software (IBM Corp., USA). The Shapiro–Wilk test was used to test the normality of the data distribution. The ANOVA test was used to test the differences between groups following different diets for data following a normal distribution (pHDI scores) and the Kruskal–Wallis test for data deviating from a normal distribution (Nutritional knowledge; Iron intake). A *t*‐test was used to compare differences in the pHDI index between the sexes, while a Mann–Whitney test was used to compare iron intake and nutritional knowledge between the sexes. Pearson's *χ* ^2^ test was used to compare qualitative variables, including links between gender and dietary patterns, as well as between dietary patterns and the use of dietary supplements. Correlations between quantitative data, including BMI, pHDI scores, nutrition knowledge scores, and iron intake were investigated using Spearman's test. The study's defined significance level was set to *α* = 0.05.

## 3 Results

### 3.1 Participant Characteristics

The participants had a median age of 29 (18–62) years, a mean body mass of 70 ± 13 kg, a mean height of 173 ± 8 cm, and a median BMI of 22.96 (16.38–32.03) kg/m^2^. Participants were divided into three dietary groups: vegans (VEG, *n* = 36), lactovegetarian group (LOW, *n* = 10), including individuals following lacto‐ (*n* = 5), ovo‐ (*n* = 2), or lacto‐ovo (*n* = 3) vegetarian diets, and omnivores (OMN, *n* = 54). No significant differences between the groups were found in height, body mass (*p* > 0.05, ANOVA test), age, or BMI (*p* > 0.05, Kruskal–Wallis test). No significant association was found between sex and the dietary model applied (*p* > 0.05, Pearson's Chi‐square test). Anthropometric data are presented in Table 2. The results showed variation across dietary groups in the prevalence of sports injuries over the past year. The vegan group had the highest proportion of reported injuries (*n* = 11, 30.6%), followed by the lactovegetarian group (*n* = 3, 30.0%), and the omnivorous group (*n* = 6, 11.1%).

**TABLE 2:** Anthropometric characteristics and age distribution of participants across dietary subgroups (VEG, LOW, OMN).

	Age [years]	Height [cm]	Weight [kg]	BMI [kg/m^2^]
	Mean ± SD	Mean ± SD	Mean ± SD	Mean ± SD
	Median	Median	Median	Median
	Min–Max	Min–Max	Min–Max	Min–Max
VEG	33 ± 10	172 ± 6	68 ± 11	22.84 ± 2.65
	31	172	68	22.83
	18–54	159–185	43–85	16.38–30.12
LOW	37 ± 11	175 ± 13	71 ± 16	22.85 ± 3.27
	36	172	71	23.27
	22–50	160–198	19–95	18.00–29.30
OMN	33 ± 12	172 ± 9	71 ± 13	23.88 ± 3.45
	29	172	73	23.22
	18–62	158–190	48–105	18.31–32.03
Overall	33 ± 11	173 ± 8	70 ± 13	23.4 ± 3.18
	29	172	70	22.96
	18–62	158–198	43–105	16.38–32.03
*p*‐value	*p* = 0.401	*p* = 0.612	*p* = 0.535	*p* = 0.556

*Note:* No significant differences were found between dietary groups in age and BMI (*p* > 0.05, Kruskal–Wallis test), as well as in height and weight (*p* > 0.05, ANOVA).

Abbreviations: BMI, Body Mass Index; LOW, vegans consuming milk and/or eggs and pescatarians; OMN, omnivorous group; SD, standard deviation; VEG, vegan group.

### 3.2 Motivations for Following a Plant‐Based Diet

A total of 137 responses were provided by participants from the VEG and LOW groups regarding their reasons for following a plant‐based diet. The most frequently indicated motivation was ethical reasons (*n* = 37, 27.0%), followed by health‐related reasons (*n* = 20, 14.6%) and ecological concerns (*n* = 19, 13.9%). Religious motives were reported by 3 individuals (2.2%), and 1 person (0.7%) indicated a personal dislike for the taste of meat.

### 3.3 Dietary Supplement Use

A total of 76 participants (76.0%) declared the use of dietary supplements, while 24 individuals (24.0%) reported no supplementation. A statistically significant association was found between dietary model and supplement use (*p* = 0.004, Pearson's *χ* ^2^ test). Supplement use was most prevalent in the VEG group (91.7%), followed by the LOW group (90.0%), and the OMN group (63.0%).

**FIGURE 1:**
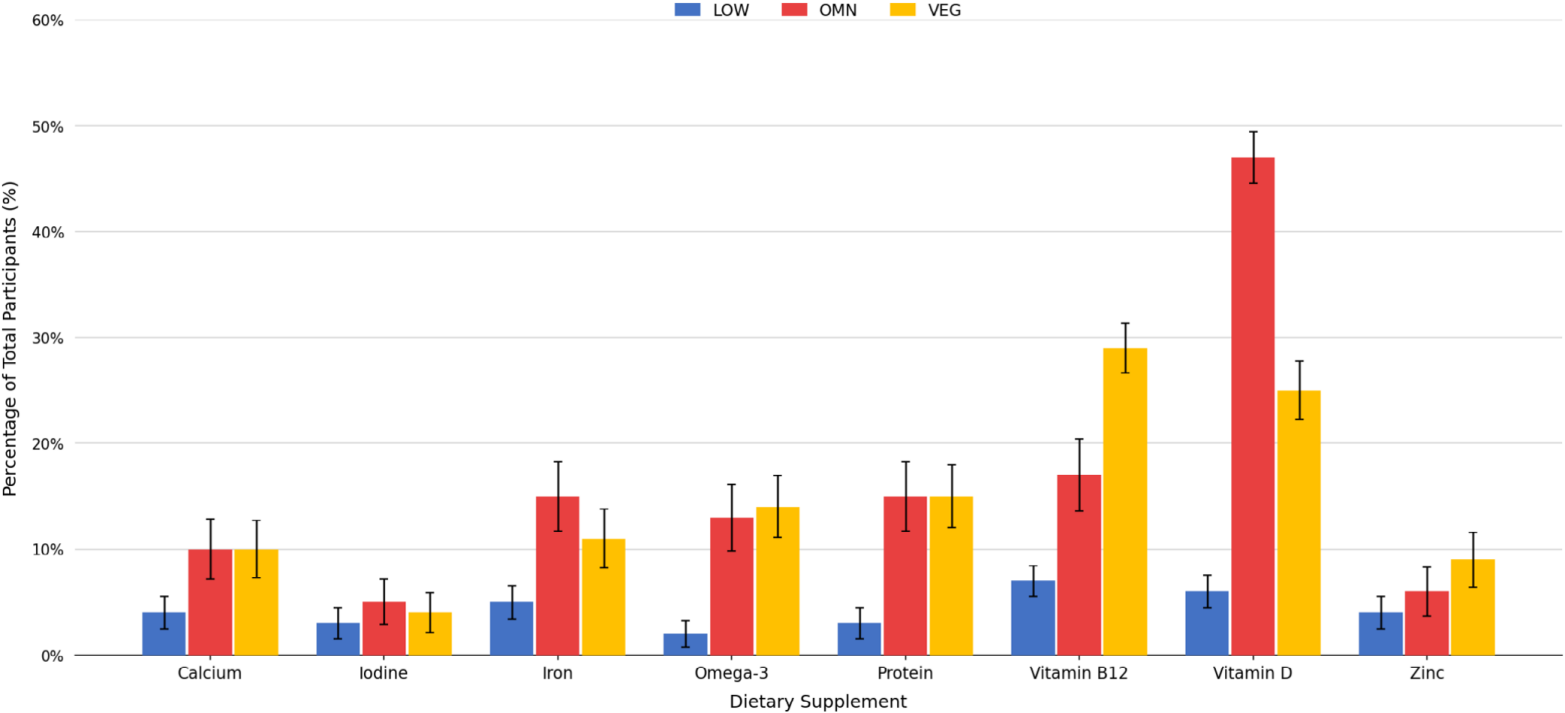
Comparison of supplement use across different dietary groups: VEG, vegan group; LOW, vegans consuming milk and/or eggs and pescatarians; OMN, omnitarian group. Error bars represent standard error (SE). Differences between dietary groups were statistically significant (*p* = 0.004, Pearson's *χ* ^2^ test).

The most commonly used supplement in the VEG group was vitamin B12 (*n* = 29), followed by vitamin D (*n* = 25) and protein (*n* = 15). In the LOW group, vitamin B12 was reported by 7 individuals, vitamin D by 6, and iron by 5. In the OMN group, vitamin D was the most frequently supplemented nutrient (*n* = 47), followed by vitamin B12 (*n* = 17), iron (*n* = 15), and protein (*n* = 15). The results are presented in Figure 1.

### 3.4 Nutritional Knowledge

The overall nutritional knowledge (NK) score across all groups was represented by a median of 8 (2–10) points, as shown in Table 3. Statistically significant differences were found between dietary groups (*p* = 0.002, Kruskal–Wallis test). Post hoc analysis revealed that the OMN group had significantly lower nutritional knowledge compared to both the VEG group (*p* = 0.044) and the LOW group (*p* = 0.004). No significant differences were found between the VEG and LOW groups (*p* = 0.336).

**TABLE 3:** Nutritional knowledge, iron intake, and the Pro‐Healthy Diet Index in the studied subgroups.

	II [mg/day]	pHDI [points]	NK [points]
	Mean ± SD	Mean ± SD	Mean ± SD
	Median	Median	Median
	Min–Max	Min–Max	Min–Max
VEG	25.22 ± 13.8	25.57 ± 7.77	8 ± 1
	20.5^a^	26.05	8^a^
	7.22–59.94	10.7‐45,0	5–10
LOW	20.15 ± 9.54	31.82 ± 10.22	9 ± 1
	19.97^b^	30.35	8^b^
	8.01–42.48	20.00–50.60	7–10
OMN	15.21 ± 10.02	24.76 ± 11.06	7 ± 2
	13.21^a^, ^b^	23.7	7^a^, ^b^
	3.93–52.13	3.7–53.9	2–10
Overall	19.31 ± 12.06	25.76 ± 10.03	7 ± 2
	16.06	25	8
	3.93‐59‐94	3.7–53.9	2–10
*p*‐value	*p* < 0.001^a^	*p* > 0.05	*p* = 0.044^a^
	*p* = 0.052^b^		*p* = 0.004^b^

*Note:* *p‐*values represent between‐group comparisons (VEG vs. LOW vs. OMN) using Kruskal–Wallis test (II, NK) and ANOVA (pHDI).

Abbreviations: II, iron intake; NK, nutritional knowledge; pHDI, pro‐healthy diet index; SD, standard deviation.

^a^ Values with the same letter significantly differ from each other.

^b^ Values with the same letter significantly differ from each other.

### 3.5 Diet Quality

The overall Pro‐Healthy Diet Index (pHDI) score was 25.76 ± 10.03. The LOW group demonstrated the highest mean pHDI score (31.82 ± 10.22), followed by VEG (25.57 ± 7.77) and OMN (24.76 ± 11.06), as shown in Table 3. No significant differences were observed between sexes or across dietary groups in pHDI scores (*p* > 0.05; *t*‐test and ANOVA, respectively).

### 3.6 Iron Intake

The median iron intake across all participants was 16.06 mg/day (range: 3.93–59.94 mg/day). Significant differences were observed between dietary groups (*p* < 0.001, Kruskal–Wallis test). Post hoc analysis revealed that iron intake was significantly higher in the VEG group compared to the OMN group (*p* < 0.001), while the difference between the LOW group and OMN was on the edge of significance (*p* = 0.052). No significant difference was observed between the LOW and VEG groups (*p* = 0.397).

### 3.7 Correlations

The pHDI was positively correlated with iron intake (*p* = 0.004, rho = 0.288) and physical activity (*p* = 0.002, rho = 0.308), and negatively correlated with BMI (*p* = 0.002, rho = −0.309, Spearman's test). No significant correlation was found between pHDI and nutritional knowledge (*p* = 0.065). Iron intake was positively correlated with nutritional knowledge (*p* = 0.014, rho = 0.246, Spearman's test). No significant correlation was found between self‐assessed nutritional knowledge and the points obtained in the nutritional knowledge test (*p* > 0.05). A significant correlation was observed between self‐assessed diet quality and pHDI score (*p* < 0.001, rho = 0.419, Spearman's test).

## 4 Discussion

The aim of the study was to conduct a comparative analysis of diet quality between recreational runners following a vegan, predominantly plant‐based diet and those adhering to an omnivorous diet in Warsaw. A particular focus was placed on comparing iron intake levels and dietary supplements intake between these three dietary groups. To the best of our knowledge, this is the first study examining the relationship between iron intake and diet quality specifically in a group of amateur runners living in urban areas, using an IRONIC‐FFQ Questionnaire. The study provided valuable insights into the nutritional habits and supplement use patterns of individuals with different dietary patterns, highlighting key differences and potential areas for nutritional intervention and education in runners.

All three hypotheses were supported by the findings. Consistent with the first hypothesis, plant‐based runners did not demonstrate poorer diet quality. No significant differences in pHDI scores were found across groups (*p* > 0.05), with the LOW group achieving the highest mean pHDI score (31.82 ± 10.22), likely reflecting additional points earned for dairy, egg, and fish consumption. In line with the second hypothesis, plant‐based runners showed iron intakes comparable to or higher than those of omnivores. Vegan and lactovegetarian runners demonstrated significantly higher iron intakes (*p* < 0.001), with median intakes of 20.5 mg/day and 19.97 mg/day, respectively, compared with 13.21 mg/day in the OMN group. The third hypothesis was also confirmed: supplement use was significantly more prevalent among plant‐based groups (VEG: 91.7%, LOW: 90.0%) than among omnivores (63.0%, *p* = 0.004, *χ* ^2^ test), supporting the notion that vegan runners actively use supplementation.

The motivations for adopting a plant‐based diet among participants from the VEG and LOW groups were diverse, with ethical concerns emerging as the most common reason (27.0%). Health‐related motivations (14.6%) and ecological concerns (13.9%) were also frequently cited, indicating that many individuals are driven by a combination of personal health goals and broader environmental or moral considerations. A small number of participants cited religious beliefs (2.2%) or a personal dislike for the taste of meat (0.7%) as influencing their dietary choices. These findings align with existing studies, which highlight the multifactorial nature of dietary decision‐making and suggest that ethical and health‐related motivations often play a central role in plant‐based diet adoption (Janssen et al. 2016).

Using a similar technique, Gacek et al. (2024) examined the diet quality among female athletes practicing team sports. The findings showed that both the pro‐Health Diet Index (pHDI10) and the non‐Healthy Diet Index (nHDI14) scored at low levels, with a mean pHDI of 17.70 points. This indicates that their eating patterns neither maximized health promoting foods nor minimized less healthy choices, which is consistent with our findings of relatively low diet quality across all groups. However, unlike the present study, research by Gacek et al. did not differentiate athletes by dietary model, limiting conclusions about the role of dietary pattern in determining diet quality among physically active individuals. Studies examining diet quality across dietary patterns consistently show higher diet quality among vegetarians and vegans compared with omnivores (Clarys et al. 2014; Groufh‐Jacobsen et al. 2023).

In the study by Jedut et al. (2023) long‐term vegetarians had higher diet quality than their omnivorous counterparts, which is consistent with our observation of comparable or higher pHDI scores among plant‐based runners. Despite better diet quality, vegetarians in their study showed a higher prevalence of vitamin B12 and vitamin D deficiencies and elevated homocysteine levels. Those findings suggest that higher diet quality scores do not necessarily guarantee adequate micronutrient status. While micronutrient status was not assessed in the present study, the high prevalence of vitamin B12 and vitamin D supplementation observed among our plant‐based runners may indirectly reflect an awareness of these risks. It should be noted, however, that the study by Jedut et al. was conducted in a general, non‐athletic population with a relatively small sample, which considerably limits the comparability of their findings to our athletic sample.

An interesting counterpoint comes from Kwaśniewska et al. (2023) who examined dietary antioxidant intake and diet quality across omnivores, flexitarians, and vegetarians in a large sample of adults. Omnivores demonstrated significantly higher intake of natural antioxidants including vitamins C, E, and zinc compared to flexitarians, while flexitarians showed a diet high in total fat and low in fiber despite reduced meat consumption. Although this study focused on dietary habits rather than diet quality itself, dietary habits are often closely linked to diet quality, as they reflect consistent food choices over time. These findings suggest that adopting a plant‐based diet does not automatically guarantee better nutritional intake, which aligns with the relatively low pHDI scores observed across all groups in our study. However, direct comparison is limited as the study by Kwaśniewska et al. used antioxidant intake as a measure of diet quality rather than a validated diet quality index.

### 4.1 Iron Intake

The present study demonstrated significant differences in iron intake between dietary groups following different nutritional patterns among amateur runners. The Estimated Average Requirement (EAR) in Poland used as the reference standard is 6–8 mg/day for females (depends on age) and 6 mg/day for males (Głąbska et al. 2017). Overall, many participants, regardless of dietary pattern, met or exceeded the EAR for iron. Notably, all male VEG and LOW met or exceeded the Average Requirement (EAR), suggesting that plant‐based diets, when well‐planned, can effectively meet iron requirements.

It should be noted, however, that athletes have higher iron needs compared to the general population (Dawczynski et al. 2022; Fernandes et al. 2024; Kardasis et al. 2023). This is due to increased physical demands and iron loss through sweat and exercise‐induced stress (Sinclair and Hinton 2005; Solberg and Reikvam 2023). Athletes who are at particular risk of iron loss, including distance runners, should aim to consume higher amounts of iron than the RDA standards (men > 8 mg/day; women > 18 mg/day) (Nebl et al. 2019). When iron intake was assessed against athlete‐specific recommendations, a higher proportion of female omnivorous runners fell below the RDA compared to plant‐based groups (*n* = 18; 18%).

Among male runners, the proportion not meeting athlete‐specific recommendations was generally low across all groups, with a slight decline observed in the OMN group when applying athletic rather than general population standards (*n* = 8; 8%). Plant‐based males maintained iron adequacy regardless of the reference standard applied, while female plant‐based runners showed only minor shortfalls (*n* = 2; 2%). These findings suggest that while plant‐based diets can support adequate iron intake, even among athletes, female OMN may be particularly at risk of insufficient consumption when higher physiological demands are considered. This underscores the importance of dietary planning and monitoring, especially in female endurance athletes, to ensure iron needs are consistently met.

A study done by Woodbridge et al. (2020) assessing the dietary intake of vegan runners, using 3‐day food diaries, found several deficiencies across different macronutrients and micronutrients. Interestingly, the study reported that the average iron intake among the vegan runners was above the Reference Nutrient Intake (RNI). However, when examined at the individual level, 87% of female vegan runners fell below the recommendations, suggesting that the group mean was skewed upward by a small number of participants using iron supplements. This pattern mirrors our findings, where plant‐based groups achieved higher median iron intakes than omnivores, yet a notable proportion of female participants still failed to meet athlete‐specific recommendations, particularly in the OMN group.

Beermann et al. (2020) investigated nutritional intake and energy availability in collegiate distance running. Regarding iron, all male participants and 75% of female participants met the RDA, although 24 runners were taking iron supplements, highlighting a possible reliance on supplementation to achieve adequate intake. Dobrowolski and Włodarek (2020) assessing iron intake among female football players, found that iron consumption was marginally sufficient, with average intake levels just exceeding the EAR. Similarly, the study by Fujii et al. (2015) examined iron intake and anemia status among college athletes across various sports. The findings revealed that 83% of male and 15% of female athletes consumed more iron than the RDA. While most participants met general population EAR values, applying athlete‐specific RDA standards revealed that female runners, particularly omnivores, were most at risk of inadequate iron intake. Our findings reinforce the need for routine iron status monitoring and strategic dietary planning in athletic populations, especially among females adhering to plant‐based diets.

### 4.2 Nutritional Knowledge and Its Connection to Iron Intake

Attention should be paid to the relationship between nutritional knowledge and iron intake (*p* = 0.014, rho = 0.246, Spearman's test). The OMN group scored significantly less points compared to the VEG (*p* = 0.044) and LOW (*p* = 0.004) groups. This suggests that individuals with better nutritional knowledge are more likely to recognize the importance of iron, particularly in the context of endurance performance and anemia prevention, and to take dietary measures accordingly, such as incorporating iron‐rich foods or using supplements.

The findings of Leonard et al. (2014) complement our observations regarding the relationship between nutrition knowledge and dietary iron intake. In the study done on women, a positive correlation was identified between iron‐specific nutrition knowledge and dietary iron intake, although this did not directly translate to meeting the RDI. Additionally, significantly lower serum ferritin levels were found in vegetarians compared to non‐vegetarians. Similarly, our data revealed that participants with higher nutrition knowledge scores tended to consume more iron, particularly those following plant‐based diets. It should be noted, however, that higher dietary iron intake in plant‐based runners does not necessarily reflect adequate iron status, as serum ferritin levels were not assessed in the present study.

### 4.3 Supplement Use

The differences between individuals following different dietary models and dietary supplementation were also demonstrated (*p* = 0.004, Pearson's *χ* ^2^ test). Supplement use was more prevalent in the plant‐based groups. Nearly all individuals adhering to vegetarian or vegan diets engaged in supplementation (91.7% of VEG and 90.0% of LOW), whereas over one‐third of omnivores (37.0%) reported no supplement use. This suggests that plant‐based runners are more aware in addressing potential nutritional gaps, while many omnivorous runners may rely solely on their regular diet without additional supplementation. The pattern of supplement use observed in our study is consistent with broader evidence on supplementation practices in plant‐based athletes. Vitamin B12 and vitamin D were the most supplemented nutrients among vegan and lactovegetarian runners, reflecting well‐documented risks of deficiency associated with plant‐based diets (Dawczynski et al. 2022; Fernandes et al. 2024). Vitamin D supplementation was the most frequently used supplement among all dietary groups, indicating a broad recognition of vitamin D's importance.

Any dietary pattern, if not properly balanced, may increase the risk of nutrient deficiencies. A study by Woodbridge et al. (2020) assessing nutrient status in vegan runners found deficiencies in certain macronutrients, as well as in vitamin D and selenium. Findings of Schüpbach et al. (2017) examining micronutrients intake and status in omnivores, vegetarians, and vegans found deficiencies in all dietary groups. Individuals following an omnivorous diet had a low intake of magnesium, vitamin C, vitamin E, niacin, and folate. In contrast, those adhering to a vegan diet reported inadequate calcium intake and only marginal levels of vitamins D and B12. Both omnivorous and plant‐based diets can be lacking in essential nutrients if not properly planned (Neufingerl and Eilander 2021). Therefore, understanding the potential nutritional gaps in a vegan diet is important for creating well‐balanced meal plans and identifying when supplementation may be necessary. However, it is difficult to determine whether the study participants were taking supplements appropriately. This study focused on dietary quality and iron intake, not the consumption of individual nutrients, including vitamins and minerals. Therefore, it is difficult to determine whether the study group experienced any deficiencies that would require appropriate supplementation. As studies indicate, the overconsumption of dietary supplements is common in both the amateur and professional athlete population (Garthe and Maughan 2018; Lun et al. 2012; Maughan et al. 2018). It should be noted that despite relatively high supplement use across all groups, the pHDI score indicated an overall low intensity of health‐promoting dietary characteristics. This raises an important concern, as rather than addressing underlying diet inadequacies, participants may have been relying on supplements as a substitute for a well‐balanced diet. Improving overall diet quality should therefore be the primary focus for amateur runners, rather than relying on supplements to fill nutritional gaps.

While the study provides novel insights into the dietary patterns of physically active individuals, several limitations should be acknowledged. The use of the KomPAN questionnaire, which relies on self‐reported food frequency data, may introduce recall bias and inaccuracies in estimating habitual intake. Additionally, the study utilized a non‐random, convenience sample, which limits the representativeness of the findings and restricts their generalizability to the broader adult population. The cross‐sectional design further constrains the ability to establish causal relationships between dietary behaviors and health outcomes. Despite these limitations, a key strength of the study lies in its focus on amateur runners, a relatively understudied group, providing valuable data on their dietary habits, iron intake, and supplementation practices. These methodological considerations should be acknowledged when interpreting the findings and designing future research in this area.

In conclusion, this study highlights that amateur runners following plant‐based diets, including vegan and lacto‐ovo vegetarian patterns, can achieve adequate iron intake and comparable or even higher diet quality than omnivores, provided their diets are well‐planned. Supplement use was considerably more prevalent among plant‐based runners, reflecting a proactive awareness of potential nutritional gaps associated with restrictive dietary patterns. However, given the relatively low pHDI scores observed across all groups, dietary quality improvement should remain the primary focus rather than reliance on supplementation alone.

The findings of the present study have several practical implications for athletes, coaches, and nutrition practitioners. Amateur runners following plant‐based diets should be encouraged to focus on consuming iron‐rich plant foods alongside absorption enhancers such as vitamin C, while limiting inhibitors such as phytates and calcium during iron‐rich meals (Nolte et al. 2024; Piskin et al. 2022). Sports nutritionists working with plant‐based runners should pay particular attention to female athletes, who appear most vulnerable to inadequate iron intake, and should consider recommending routine blood iron status monitoring (Zourdos et al. 2015). Given the relatively low diet quality scores observed across all groups, nutrition education interventions targeting amateur runners, regardless of dietary pattern, may be beneficial in improving overall dietary quality and reducing reliance on supplementation.

## 5 Conclusion

Ultimately, these findings emphasize the importance of targeted nutrition education addressing specific micronutrient concerns relevant to different dietary patterns. Enhancing awareness around iron and other essential nutrients can empower athletes to make informed choices that optimize both health and athletic outcomes. Nutrition interventions should also consider motivational factors unique to each dietary group, aiming to bridge knowledge gaps and encourage proactive nutrient management across the athletic population. Several directions for future research emerge from these findings. Longitudinal studies examining the long‐term impact of plant‐based diets on iron status and athletic performance in amateur runners are needed. Future research should also include biochemical assessment of iron status, including serum ferritin and hemoglobin levels. Additionally, studies examining supplementation practices in this population, as well as the role of nutrition education interventions in improving dietary quality among amateur runners, would provide valuable insights.

## Author Contributions

**Gabriela Lewandowska:** writing – original draft, methodology, investigation, conceptualization, visualization, formal analysis, project administration, resources, data curation. **Hubert Dobrowolski:** supervision, writing – review and editing, formal analysis, software, methodology, validation, writing – original draft, resources.

## Conflicts of Interest

The authors declare no conflicts of interest.

## Data Availability

The data that support the findings of this study are available from the corresponding author upon reasonable request, by contacting h.dobrowolski@vizja.pl or gabriela.elagl@gmail.com.
